# Nano-electro-mechanical pump: Giant pumping of water in carbon nanotubes

**DOI:** 10.1038/srep26211

**Published:** 2016-05-19

**Authors:** Amir Barati Farimani, Mohammad Heiranian, Narayana R. Aluru

**Affiliations:** 1 Department of Mechanical Science and Engineering, Beckman Institute for Advanced Science and Technology, University of Illinois at Urbana-Champaign, Urbana, Illinois 61801, USA.

## Abstract

A fully controllable nano-electro-mechanical device that can pump fluids at nanoscale is proposed. Using molecular dynamics simulations, we show that an applied electric field to an ion@C60 inside a water-filled carbon nanotube can pump water with excellent efficiency. The key physical mechanism governing the fluid pumping is the conversion of electrical energy into hydrodynamic flow with efficiencies as high as 64%. Our results show that water can be compressed up to 7% higher than its bulk value by applying electric fields. High flux of water (up to 13,000 molecules/ns) is obtained by the electro-mechanical, piston-cylinder-like moving mechanism of the ion@C60 in the CNT. This large flux results from the piston-like mechanism, compressibility of water (increase in density of water due to molecular ordering), orienting dipole along the electric field and efficient electrical to mechanical energy conversion. Our findings can pave the way towards efficient energy conversion, pumping of fluids at nanoscale, and drug delivery.

Efficient energy conversion at nanoscale, e.g. from electrical to mechanical form, is a significant scientific challenge. In nanoscale devices, dynamic control of charged species under an electric field and converting the electrical input into an efficient mechanical energy is one of the important challenges^1^,^2^. Carbon-based nanomaterials such as carbon nanotubes (CNT), buckyballs and graphene sheets have been used extensively to harvest energy^2^,^3^,^4^,^5^. Among the possible energy harvesting and conversion schemes in CNTs, is to pump water by feeding different sources of input energy, e.g. pressure gradient, gravity, electric field, etc^6^,^7^,^8^. In CNTs, water transport has been shown to be fast and efficient^9^,^10^,^11^ which leads to potential applications in energy conversion, water desalination, cooling, transport and drug delivery^11^,^12^,^13^. To move fluids inside CNTs, several types of energy input including temperature gradient^14^, AC electric field^6^, rotating electric field^15^, radial breathing actuation of CNT^16^, static charge placement^17^, and translational-rotational coupling-driven motions^18^ were proposed and explored.

Recently, encapsulation of a single atom^19^, ion^20^, or a water molecule^21^ inside a buckyball has opened up exciting new opportunities to design novel devices by exploiting the isolated atom/molecule properties like its free charge and dipole moment^22^. For example, a single ion encapsulated in C60 is a new large molecule with a free charge which can be actuated by an electric field. Since electric fields are partially screened by CNTs or buckyballs, a desired electric field can be obtained by applying a stronger field in magnitude^23^, hence the electric field acts on the ion even in the presence of screening.

Insertion of an ion@C60 (with a diameter of 10 Å) inside a (9, 9) CNT (with a diameter of 12.24 Å) creates a piston-cylinder mechanism (reciprocating pump) with no molecular leakage between the ion@C60 and CNT due to a maximum clearance of only 2.24 Å (water molecule diameter is 2.75 Å). The pump design and simulation set up is shown in Fig. 1a. By applying an electric field on the ion@C60 inside a CNT system, the ion@C60 moves due to the applied field and pushes the fluid inside the CNT.

## Methods

**Figure 1:**
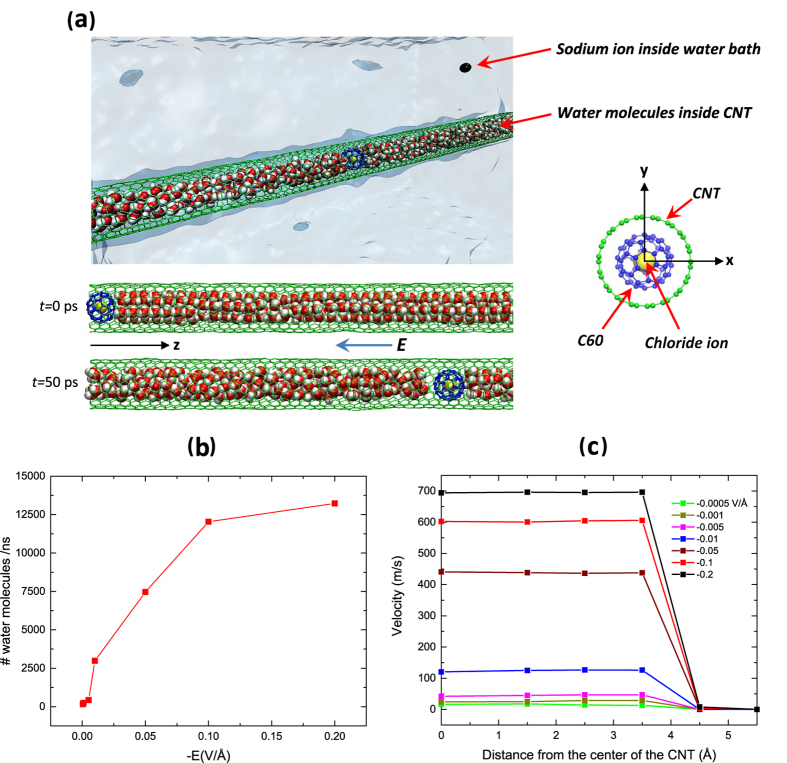
(**a**) Top left: the entire system is shown. Right: the cross-sectional view of buckyball, ion and (9, 9) CNT is shown. Buckyball fits in (9, 9) CNT with the molecular clearance. Bottom left: snapshots of water pumping with Cl^−^@C60 piston in (9, 9) CNT cylinder. As time advances, the position of Cl^−^@C60 is shown at two time snapshots. (**b**) Flux of water molecules for different electric fields (*E*). (**c**) Velocity profiles of water in (9, 9) CNT for different electric field intensities.

Molecular dynamics simulations were performed with LAMMPS^24^ to investigate the properties of the proposed pump. First, we filled the (9, 9) CNT with water under equilibrium condition^25^. Flexible Ferguson water model^26^ is used for water-water interactions. Our studies show that the water self-diffusion coefficient using the flexible Ferguson model is equal to 2.41(×10^−5^) cm^2^/s which is close to the experimental value of 2.3(×10^−5^) cm^2^/s for bulk water^25^. For carbon-water interactions, we used the force field developed in our group^27^. For C-C bonded interactions, we used the Tersoff potential with the Lindsay-Broido correction^28^. This potential is able to predict the thermal/mechanical properties of both CNT and graphene close to the experimental values. After filling the CNT, the bath was removed and we further equilibrated the water filled CNT for 1 ns. The CNT length was chosen to be 15 nm for all the simulations. Periodic boundary condition was applied in all the three directions. A chloride ion was placed in the center of a C60 and the ion@C60 complex was placed in the CNT by deleting a few water molecules. Finally, the resulting complex was placed in a bath of water (8 × 8 × 15 nm) containing a sodium ion neutralizing the system (Fig. 1a). The system was equilibrated for another 2 ns. The non-bonded C-C interaction LJ parameters are ɛ = 0.0553 kcal/mol, and σ = 3.4 Å^23^. For the ion interactions, we used the mixing rules in which the LJ parameters for Cl-Cl and Na-Na interactions are ɛ = 0.01279 kcal/mol, σ = 4.8305 Å and ɛ = 0.3526 kcal/mol, σ = 2.1600 Å, respectively^29^. The simulation was performed in NVT ensemble. Temperature was maintained at 300 K by applying the Nosè-Hoover thermostat^30^,^31^ with a time constant of 0.1 ps. The cutoff distance for the LJ interactions was 15 Å. The long-range electrostatic interactions were computed by using the Particle-Particle-Particle-Mesh method (PPPM)^32^. We applied electric fields ranging from *E = *−0.0005 to −0.2 V*/*Å in the axial direction (z) of the CNT. During the simulations, the sodium and chloride ions do not collide with each other (see the supplementary information). In Fig. 1a, snapshots of the pumping of water and the translocation of Cl^−^@C60 are shown for *E = *−0.1 V*/*Å.

## Results and Discussion

To estimate the pumping power of the system, we measured the flux of water versus the electric field intensity (Fig. 1b). For very low electric field intensities (*E* = −0.0005 V*/*Å), we observed the flux to be 168 water molecules/ns which shows that even for small values of *E*, the water flux is still high. By increasing the electric field intensity from *E = *−0.0005 V*/*Å to *E = *−0.1 V/Å, a sharp increase in flux is observed (Fig. 1b). A further increase in the electric field from *E = *−0.1 to −0.2 V*/*Å does not give rise to a significant change in flux. To investigate the type of the flow and the hydrodynamic nature of the flow inside the CNT, the velocity profiles in (9, 9) CNT are computed for electric field intensities ranging from *E = *−0.0005 to −0.2 V*/*Å (Fig. 1c). For all electric field intensities, the velocity profile is plug-like. Interestingly, in contrast to gravity driven flows, the flow transitions into steady-state quickly for our electric field driven pump (transition time for the electric field driven flow is 500 ps, and for a gravity driven flow is 4–5 ns)^11^. The velocity of the flow reaches a very large value of 700 m/s for *E = *−0.2 V*/*Å. For a lower *E = *−0.05 V*/*Å, the velocity is 440 m/s which is still a large velocity compared to the flows generated in other electric field driven flows^11^. The velocity variation with the electric field is not linear for high electric fields (see the supplementary information for details). To visualize the fast pumping of water in the CNT, Video 1 is provided as a supplementary material. As the electric field intensity increases, the fluctuation/oscillation of the water dipole (around x and y directions) decreases as the axial component of the force which is applied on the hydrogen and oxygen atoms gets stronger (note that the summation of forces on each water molecule is zero). In Fig. 1c, for r >4 Å (r is the distance from the center of the CNT), a region with water density less than 5% is observed (depletion region). For larger electric fields, the axial velocity decreases sharply to zero near the CNT wall whereas for lower electric fields velocity decreases with a smaller slope near the CNT wall. This phenomenon is due to the structured organization of water molecules in higher electric fields that will be discussed later in this paper.

**Figure 2:**
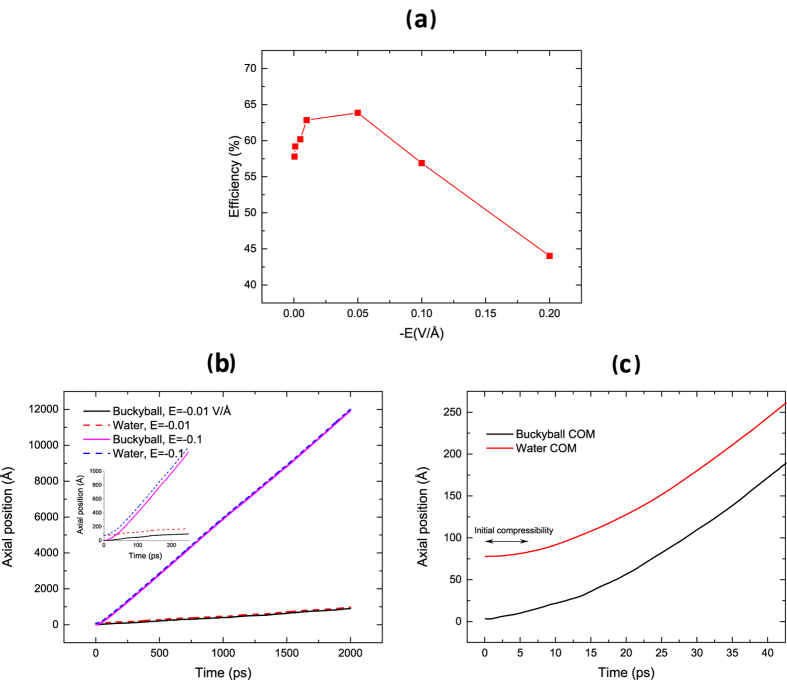
(**a**) Efficiency of the water pump as a function of the external electric field. (**b**) Axial position of the buckyball and water COM is shown for *E* = −0.01 and −0.1 V/Å (note that the initial axial positions of the buckyball and water COM are 3.5 and 77.5 Å, respectively). (**c**) Initial compression of water molecules with buckyball movement for *E* = −0.2 V/Å.

The high velocities are due to the highly efficient conversion of electrical to mechanical energy. The conversion efficiency is defined as the ratio of the extractable output mechanical power (*P*_*m*_) to the input electric power (*P*_*e*_). The input is simply the work done per unit time by the electric field on both ions. The input electric power can be written as


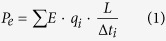


where *q*_*i*_ is the charge of the ion *i*, *L* is the length of the simulation box in z, and Δ*t*_*i*_ is the time interval the ion travels the distance *L*. To investigate how much mechanical energy can be extracted from the flow of water molecules, different external gravities are applied to the water molecules in the absence of electric fields. Then the gravity force that produces the same water flow rate as the flow generated by the electric field, is selected. Thus, the mechanical power (*P*_*m*_) is found by


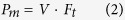


where *V* is the velocity of the center of mass (COM) of the water molecules and *F*_*t*_ is the total gravity force on the water molecules. The efficiencies (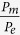) are plotted in Fig. 2a versus the applied electric fields. The energy conversion efficiency first increases with the applied electric field and then decreases for the high fields of −0.1 and −0.2 V/Å. For these high electric fields, the water velocity reaches a saturation state (see Fig. 1b,c); therefore, the water velocity does not increase comparably for a given increase in the electric field which, in return, reduces the efficiency. The best efficiency of the proposed pump is 64% for *E = *−0.05 V/Å. This value is about 5.3 times higher than the maximum efficiency of 12% for electrokinetic energy conversion systems^6^,^33^.

The axial position of the COM of the buckyball and the COM of all water molecules is tracked versus time for *E = *−0.01 and *E = *−0.1 V/Å (Fig. 2b). At the beginning of each simulation, water COM does not move for 5–10 ps due to the compressibility of water while the buckyball COM moves along the z axis (see Fig. 2c,b inset). We observed that the initial movement of the water COM is hindered for higher electric fields. This results in a depletion region around Cl^−^@C60. The depletion region grows as electric field intensity, *E*, increases. After a short period of compression (~10 ps), both the buckyball and water move in the axial direction linearly with time (Fig. 2c). The slope of the position-time curve increases with the electric field. The high compressibility of water is found to be related to the organized arrangement of water molecules under electric fields.

**Figure 3:**
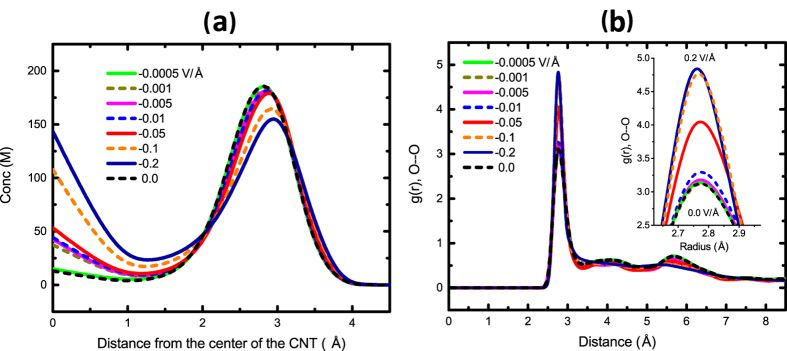
(**a**) Density distribution of water in the radial direction of the CNT for different electric field intensities (*E*). (**b**) Average radial distribution function (RDF) of oxygen-oxygen atoms of water molecules inside the CNT for different electric field intensities (*E*).

To investigate how the structure and orientation of water molecules are affected by the applied electric field, the radial density profile of water is computed (Fig. 3a). As the electric field increases, the first density peak (near the CNT wall) decreases. For higher electric fields, another density peak in the center of the CNT is observed. The second density peak is due to the compression of water molecules. For *E = *−0.2 V/Å, there is a shift in the first density peak location and the density variation is also sharper. The sharpness and shift of the first density peak can be explained by the structural arrangement of water molecules in electric fields. Water has a hexagonal structure (ice-like) in a (9, 9) CNT^25^ with a strong hydrogen bond (HB) network. Once an electric field is applied, water dipole orients in the field and the initial HB network in the (9, 9) CNT is disordered.

**Figure 4:**
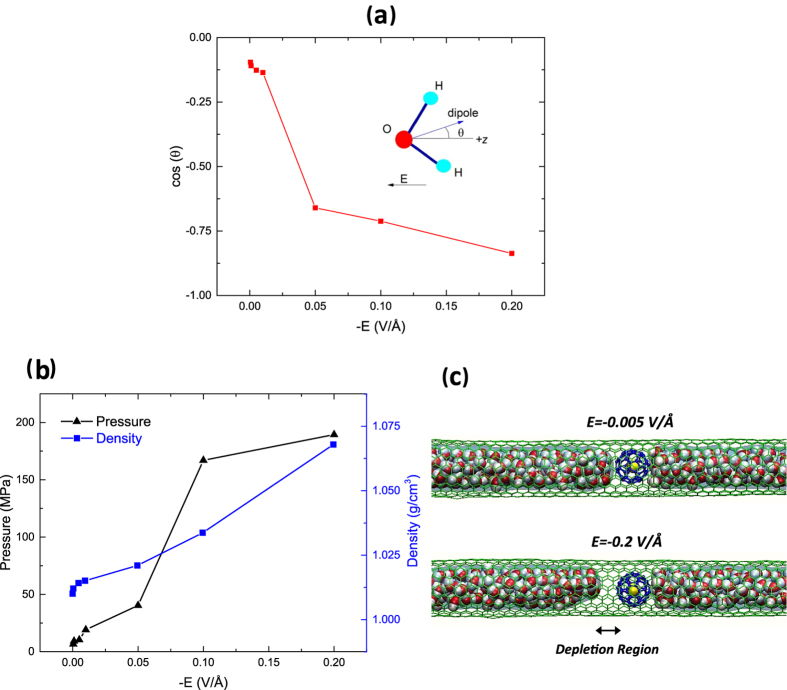
(**a**) Averaged orientation of the water dipole with respect to the axial direction of CNT (+*z*) for different electric field intensities (*E*). (**b**) (Left axis): pressure of the water molecules for different electric field intensities (*E*). (Right axis): density of the water molecules and their compressibility for different electric field intensities. (**c**) Snapshots of the simulations for *E = *−0.005 and *E = *−0.2 V/Å where there exists a depletion region around C60 for the higher electric field due to the compressibility of water.

To further study the rearrangement and structure of water molecules in strong electric fields, we computed the radial distribution function (RDF) of oxygen atoms inside the CNT for different applied electric fields (Fig. 3b). The first coordination shell becomes sharper and increases with the applied electric field, implying that more water molecules are found in the first coordination shell for higher electric fields. The increase in the first peak of RDF can be explained by the orientation of the dipole in the field. We computed the average orientation of the water dipole with respect to the axial direction of the CNT, *z*, for different *E* (Fig. 4a). The dipole oscillation about the z axis decreases as *E* increases. For *E = *−0.2 V/Å, the dipole is strongly oriented along the z axis. For *E = *−0.0005 to −0.05 V/Å, the dipole angle variation with respect to the z axis is large, giving rise to higher noise and less unidirectional transport.

Another interesting observation is the compressibility of water during the energy conversion process. The compressibility of water for different electric fields is computed by counting the number of water molecules and the volumetric space occupied (right axis of Fig. 4b). For *E = *−0.2 V/Å, water is compressed up to 1.07 times its bulk value. The compression is due to the large initial acceleration of the buckyball. Aligned water dipoles cannot make as many hydrogen bonds as they do in the case of no electric field (because of geometrical necessities required for maximum number of HB in bulk water, e.g. tetrahedron). Consequently, water molecules get closer to each other, giving rise to higher compressibility in the case of an applied electric field. The compressibility signature can be seen by the depletion region induced around C60 (Fig. 4c). As shown in Fig. 3a, the electric field tends to fill the central region of the CNT to further compress water.

We computed the pressure induced due to the applied electric-field by using the following expression


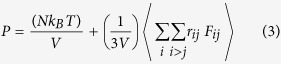


where, *N* is the number of atoms, *k*_*B*_ is the Boltzmann constant, *T* is the temperature, *V* is the volume that water molecules occupy inside the CNT, *r*_*ij*_ and *F*_*ij*_ are the distance and force between atoms *i* and *j* in the system, respectively. The induced pressure for different applied electric fields reveals that the pressure increases monotonically for electric field ranging from −0.0005 to −0.2 V/Å (Fig. 4b (left axis)).

## Conclusions

In summary, we proposed and studied a novel nano-electro-mechanical pump with an efficiency as high as 64% for electro-mechanical conversion of energy. Ion@C60-CNT can pump water molecules with a considerable amount of flux (13,000 water molecules/ns). Our nano-pump benefits from the ordered transport of water molecules under electric fields. Such a design can be useful in the development of molecular pumps, transport of water in carbon nanotubes, nanoscale heat transfer pumps, and drug delivery applications.

## Additional Information

**How to cite this article**: Farimani, A. B. *et al.* Nano-electro-mechanical pump: Giant pumping of water in carbon nanotubes. *Sci. Rep.* **6**, 26211; doi: 10.1038/srep26211 (2016).

## Supplementary Material

Supporting Information
