# Supplementary material for: Nano-electro-mechanical pump: Giant pumping of water in carbon nanotubes
Source: Sci Rep. 2016 May 19;6:26211. doi: 10.1038/srep26211 (PMC4872148; doi:10.1038/srep26211)
Supplement: Supporting Information [file srep26211-s1.doc]

Supplementary Information

Nano-electro-mechanical pump: Giant pumping of water in carbon nanotubes

Amir Barati Farimani, Mohammad Heiranian, Narayana R. Aluru

Department of Mechanical Science and Engineering
Beckman Institute for Advanced Science and Technology
University of Illinois at Urbana-Champaign, Urbana, Illinois 61801

**Na^+^ and Cl^-^ interaction**

In all the simulations, the sodium ion moves in the direction opposite to that of the chloride ion without any collision. This is because the external electric field dominates the induced electric field between the two ions. The distance between the two ions are plotted during the simulation for two cases of low and high electric fields (Figure S1). As shown, the two ions always keep a minimum distance of 35 Å.

Figure S1. Distance between the sodium and chloride ions as a function of a time for E=-0.005 and -0.2 V/Å.

**Electric field-velocity relationship**

The applied electric field as a function of the velocity of water in the CNT is plotted in Figure S2. As shown, for low electric fields (-E<0.05 V/Å), the field-velocity relationship is quite linear with a velocity exponent of 1.3. However, for the electric fields higher than -0.05 V/Å, the exponent is 2.8 which is similar to the velocity-drag relationship at high speeds in the classical fluid dynamics where depending on the velocity we could have linear, quadratic, cubic and even higher order drag-velocity relationships.

Figure S2. Applied electric field as a function of average velocity of water molecules inside the CNT.
